# New *Bacillus paralicheniformis* strain with high proteolytic and keratinolytic activity

**DOI:** 10.1038/s41598-024-73468-8

**Published:** 2024-09-30

**Authors:** Saniya Aktayeva, Bekbolat Khassenov

**Affiliations:** 1 National Center for Biotechnology, 13/5 Korgalzhyn Road, 010000 Astana, Kazakhstan; 2 Faculty of Natural Sciences, L.N. Gumilyev Eurasian National University, 2 Kanysh Satpayev Street, 010008 Astana, Kazakhstan

**Keywords:** *Bacillus paralicheniformis* T7, Proteases, Keratinases, Proteases, Industrial microbiology

## Abstract

*Bacillus paralicheniformis* T7, which exhibits high proteolytic and keratinolytic activities, was isolated from soil in Kazakhstan. Its secreted proteases were thermostable and alkaline, demonstrating maximum activity at 70 °C and pH 9.0. The proteases and keratinases of this strain were sensitive to Ni^2+^, Co^2+^, Mn^2+^, and Cd^2+^, with Cu^2+^, Co^2+^ and Cd^2+^ negatively affecting keratinolytic activity, and Fe^3+^ ions have a strong inhibitory effect on proteolytic and keratinolytic activity. Seven proteases were identified in the enzymatic extract of *B*. *paralicheniformis* T7: four from the serine peptidase family and three from the metallopeptidase family. The proteases hydrolyzed 1 mg of casein, hemoglobin, gelatin, ovalbumin, bovine serum albumin, or keratin within 15 s to 30 min. The high keratinolytic activity of this strain was confirmed through the degradation of chicken feathers, horns, hooves, wool, and cattle hide. Chicken feathers were hydrolyzed in 4 days, and the degrees of hydrolysis for cattle hide, wool, hoof, and horn after 7 days of cultivation were 97.2, 34.5, 29.6, and 3.6%, respectively. During submerged fermentation with feather medium in a laboratory bioreactor, the strain secreted enzymes with 249.20 ± 7.88 U/mL protease activity after 24 h. Thus, *B. paralicheniformis* T7 can be used to produce proteolytic and keratinolytic enzymes for application in processing proteinaceous raw materials and keratinous animal waste.

**Supplementary Information:**

The online version contains supplementary material available at 10.1038/s41598-024-73468-8.

## Introduction

Proteases hydrolyze proteins via peptide bonds and are classified into serine, cysteine, aspartate, and metalloprotease families^1^. They are found in all living organisms, including microbes, animals, and plants. *Bacillus* has a high capacity for extracellular protease secretion that is active at neutral and alkaline pH; therefore, it has considerable potential as a microbial protease source^2,3^. *Bacillus* strains capable of producing proteases can grow on inexpensive nutrient media, which is important because media components cost up to 40% of the cost of the enzyme itself^4^. Bacillary proteases are tolerant to high temperatures, organic solvents, and detergents^5^, which is one reason for their use in detergents. Furthermore, these proteases are used in the food industry^6^, cheese making^7^, baking^8^, pharmaceutical industry, stereoisomeric compound synthesis^9^, cosmetics, leather industry^10^, wastewater treatment, and feed industry^11^. Most proteolytic enzymes secreted by *Bacillus* have broad substrate specificity and can hydrolyze phosphoproteins (such as caseins) and albumins (including bovine serum albumin (BSA) and ovalbumin). Of particular interest are proteases capable of hydrolyzing fibrillar proteins (such as collagen, myosin, and keratin), which are present in large quantities in animal husbandry and meat processing industries.

Feathers are composed of more than 90% proteins, including keratin, azelon, and other insoluble proteins^12^. Keratin is a structural protein densely packed into a β-sheet polypeptide chain intensely cross-linked by disulfide bonds and stabilized by non-covalent interactions^13^. According to their secondary structure, proteins of the keratin family are divided into two groups: α-keratins, which have a conformation in the form of tight coils around the long axis of the molecule, known as α-helix (the basis of mammalian hair, wool, horns, claws, and hooves), and β-zigzag polypeptide chains known as β-sheets (claw bases, lizard scales, turtle shells, feathers, beaks, and claws of birds)^14^. The abundance of disulfide and hydrogen bonds, as well as hydrophobic interactions, provides a keratin structure that is resistant to protease action^15^. Keratin is difficult to cleave using known proteases such as pepsin, trypsin, and papain^16^, and specific proteases with keratinolytic activity are required for its hydrolysis. Keratinases are proteases that can cleave keratin and belong to a family of serine proteases or metalloproteases^17^. In practice, keratinases have an advantage over proteases because they can render feathers an inexpensive feed, amino acid, and peptide source^18^. Enzymatic hydrolysis optimally retains the nutritional content of the products and greatly enhances their solubility and digestion. Recently, many studies have focused on keratinases isolated from *Bacillus*^19–23^. Nondirected mutagenesis is used to create mutant strains with enhanced keratinase activity^24^. The high biotechnological potential of bacterial keratinases has attracted considerable attention owing to their numerous industrial applications in the development of environmentally friendly processes^25^.

Here, we describe the isolation and identification of *Bacillus paralicheniformis* T7, a strain with high proteolytic and keratinolytic activities isolated from soil. When cultured on feather medium, the proteolytic and keratinolytic features of *B. paralicheniformis* T7 were investigated qualitatively and quantitatively using biochemistry, zymography, mass spectrometry, proteomics, and spectrophotometry. Chicken feather, horn, hoof, and cattle wool hydrolysis was conducted using this strain, and the hydrolysates were studied using scanning electron microscopy. The correlation between the genome, proteome, and biochemical activities of the strain was investigated. Moreover, the possibility of obtaining an enzyme preparation through submerged fermentation with *B. paralicheniformis* strain T7 in a bioreactor was shown.

## Materials and methods

### Reagents and media

Ovalbumin was purchased from MP Biomedicals (Santa Ana, CA, USA). BSA, hemoglobin, and casein sodium salt were obtained from Sigma-Aldrich (St. Louis, MO, USA). Phenylmethylsulfonyl fluoride (PMSF), pepstatin A, ethylenediaminetetraacetic acid (EDTA), E64, and other chemical reagents were purchased from Sigma-Aldrich and AppliChem (Darmstadt, Germany). Gelatin and its constituents were purchased from Titan Biotech Ltd. (Delhi, India). Feathers were collected at the poultry farm of “CAPITAL PROJECTS LTD” LLP, located in the village of Akmol, Tselinograd district, Akmola region, Republic of Kazakhstan.

The following liquid nutrient media were used for cultivation: nutrient broth [0.5% (w/v) peptone, 0.5% (w/v) NaCl, 0.15% (w/v) yeast extract, and 0.15% (w/v) beef extract, pH 7.4] and feather broth [(g/L) NaH_2_PO_4_ (0.3), Na_2_HPO_4_ (0.35), and feather powder (7.5), pH 7.0]. The agar media used for isolating and screening the strain included nutrient agar [nutrient broth with 1.5% (w/v) agar], skim milk agar [2% (w/v) skim milk, 0. 1% (w/v) NaCl, 1% (w/v) tryptone, 1% (w/v) agar], gelatin agar [0.4% (w/v) peptone, 0.1% (w/v) yeast extract, 1.5% (w/v) gelatin, 1.5% (w/v) agar], and feather agar [(g/L) NaH_2_PO_4_ (0.3), Na_2_HPO_4_ (0.35), feather powder (7.5), and agar (15), pH 7.0].

### Isolation and identification of the microorganism

For isolation, 1 g of soil was suspended in 9 mL of 0.9% (m/vol.) NaCl and the suspension was diluted 10-fold. The third 10-fold dilution was seeded onto the agarized medium. Next, 100 µL was seeded on a nutrient agar dish and incubated at 37 °C for 48 h. The proteolytic and keratinolytic activities of the strains were tested by culturing on dishes containing milk, gelatin, and feather agar. Transparent zones around the colonies confirmed the enzymatic activity of the strain. Colony purity was checked using gram staining and light microscopy (Primo star, Carl Zeiss, Goettingen, Germany). Strains were identified by proteomic profiling of ribosomal proteins and sequencing of a conserved DNA locus, as previously described^26,27^. Proteomic analysis of the strain was performed by time-of-flight mass spectrometry with matrix-assisted laser desorption and ionization (MALDI-TOF) on a Biotyper Microflex LT instrument (Bruker Daltonics, Bremen, Germany). For sequencing, the genomic DNA of the strain was isolated using a Genomic Wizard Purification Kit (Promega, Madison, WI, USA) according to the manufacturer’s protocol. The 16 S rRNA gene fragment was amplified by PCR using universal primers 27 F and 1492R. The amplified DNA fragment was sequenced by the Sanger method^28^ using BigDye™ Terminator v3.1 (Thermo Fisher Scientific, Waltham, MA, USA) according to the manufacturer’s protocol. DNA fragments were separated using an ABI 3730xl automated sequencer (Applied Biosystems, Foster City, CA, USA). Chromatograms were analyzed and compared with the reference sequence using Vector NTI software version 11 (Thermo Fisher Scientific) and the NCBI database (http://blast.ncbi.nlm.nih.gov/Blast.cgi).

### Preparation of extracellular enzymatic extract

The enzymatic extract used to evaluate activity was obtained by first culturing the cells of the strain in 5 mL of Luria broth for 16 h at 37 °C and shaking at 170 rpm in a thermostated shaker. Next, the culture was used to inoculate 150 mL of feather broth, and the cells were cultured for 48 h under the same conditions. Subsequently, the supernatant was clarified by centrifugation at 10,000 × *g* for 10 min at 4 °C and filtered through a 0.22-µm pore-sized membrane to remove microparticles and bacterial cells. The enzymatic extract was stored on ice under aseptic conditions without freezing.

### Preparation of azo-labeled substrates

Casein was prepared from skimmed cow milk by acid precipitation, followed by drying and grinding into a fine powder. Keratin was prepared from ground chicken feathers according to a previously described protocol^29^. Azocasein and azokeratin were prepared as previously described^30^.

### Determination of the enzymatic activity of the extract

Proteolytic activity was determined according to Kembhavi et al.^31^ using azocasein as a substrate. Keratinase activity was determined as previously described^22,32^, using azokeratin as the substrate.

### Effect of temperature and pH on enzymatic activity

The effect of temperature on enzymatic activity was determined by conducting the reaction in a temperature range of 30–80 °C (with an interval of 10 °C). Maximum enzymatic activity was defined as 100%. To determine thermostability, the enzymatic extract was pre-incubated for 2 h at 50, 60, and 70 °C in 0.1 M Tris buffer (pH 8.5), and then the residual activity was measured at 60 °C in 0.1 M Tris-HCl buffer (pH 8.5). The results are expressed as a percentage relative to the activity of the extract without temperature incubation, which was taken as 100%. The enzymatic activity was measured over a pH range of 3.0 to 11.0. The following buffer systems were used: citrate buffer (pH 3.0–6.0), sodium phosphate buffer (pH 6.0–7.5), Tris-HCl (pH 7.5–9.0), and glycine-NaOH (pH 9.0–11.0). Values were converted to relative units with a maximum value of 100%.

### Effect of metal ions and protease-inhibiting chemicals on enzymatic activity

The effects of metal ions (Na^+^, K^+^, Ca^2+^, Ni^2+^, Mg^2+^, Mn^2+^, Cu^2+^, Co^2+^, Cd^2+^, Ba^2+^, and Fe^3+^) were determined by using salts: NaCl, KCl, CaCl_2_, NiCl_2_, MgCl_2_, MnCl_2_, CuCl_2_, CoCl_2_, CdCl_2_, BaCl_2_, FeCl_3_. Reducing agents [β-mercaptoethanol and dithiothreitol (DTT)], protease inhibitors [phenylmethylsulfonyl fluoride (PMSF), ethylenediaminetetraacetic acid (EDTA), pepstatin A, E64], and detergents [1% Tween 20, Triton X-100, sodium dodecyl sulfate (SDS)] on enzymatic activity were determined by adding the reagents to the reaction mixture. Thereafter, enzymatic activity was evaluated at 60 °С in 0.1 M Tris buffer (pH 8.5). Activity values were considered 100% in the absence of metal ions or chemicals.

### Zymography

The ability of the enzymatic extract to degrade various protein substrates was determined using SDS-polyacrylamide gel electrophoresis (PAGE) in a 4–10% gel copolymerized with (w/v) sodium caseinate (0.1%), BSA (0.1%), gelatin (1%), and keratin (0.7%). The enzymatic extract was added to SDS-PAGE sample buffer (125 mM Tris-HCl pH 6.8, 0.002% bromophenol blue, 4% SDS, and 20% glycerol) at a ratio (sample: buffer) of 4:6. PMSF (5 mM), EDTA (5 mM), pepstatin A (0.035 mM), and E64 (0.01 mM) were added as serine, metalloprotease, asparagine, and cysteine protease inhibitors, respectively. The samples were not boiled, and no β-mercaptoethanol or DTT was added before electrophoresis. Thereafter, the gels were washed twice for 10 min each in 2.5% Triton X-100 and incubated in 500 mM Tris-HCl (pH 8.5) at 50 °С for 20 h. Next, the gels were stained with 0.08% Coomassie Brilliant Blue G-250 (AppliChem, Darmstadt, Germany) in 20% ethanol with 1.6% orthophosphoric acid and 8% ammonium sulfate for 4 h and then clarified in MilliQ water for 3–4 h until the hydrolysis zones were completely clarified. A protein marker (Color Prestained Protein Standard, cat#P7719S; New England Biolabs, Ipswich, MA, USA) was used to determine the molecular weight.

### Hydrolysis of protein substrates

Hemoglobin, gelatin, keratin, casein, ovalbumin, and BSA were tested as substrates for hydrolysis using the enzymatic extract. Each substrate was dissolved in 1 mL 50 mM Tris-HCl (pH 8.5) to a concentration of 1 mg/mL and treated with enzymatic extract at 60 °C for 30 min with periodic sampling for analysis. A substrate without enzymatic extract treatment was used as a control. The hydrolysis products were analyzed using 12% SDS-PAGE. The gels were stained with 0.08% Coomassie Brilliant Blue R-250 (AppliChem) in 50% ethanol and 10% acetic acid. A protein marker (Color Prestained Protein Standard, cat#P7719S; New England Biolabs) was used for molecular weight determination.

### Hydrolysis of keratinous raw materials

The ability of the *B. paralicheniformis* strain T7 to degrade keratinous animal waste (chicken feathers, horns, hooves, hides, and wool) was investigated. *B. paralicheniformis* strain T7 was cultured in 15 mL of nutrient broth in a shaker incubator at 37 °C and 170 rpm for 18 h. Thereafter, 1 mL inoculum was added to glass tubes containing horn (0.777 g), hoof (1.405 g), hide (1.055 g), wool (0.254 g), and feather (0.25 g) fragments in 10 mL of sodium phosphate buffer (pH 7.0). Next, the samples were degraded in a shaker incubator at 37 °C and 250 rpm for 7 days. The degree of degradation of the samples was determined by measuring the dry weights of unhydrolyzed samples. For this purpose, the culture was passed through a pre-weighed filter paper, and the remaining residue was washed twice with distilled water and dried at 60 °C to a constant weight. The results are expressed as a percentage of the initial weight of the samples, which was taken as 100%.

### Scanning electron microscopy of feather hydrolysis products

Feather samples were inoculated with 1 mL of sterile enzymatic extract in 10 mL of 0.1 M Tris-HCl buffer (pH 8.5) supplemented with 0.02% sodium azide. Incubation was conducted for 2–7 d at 37 °C. To evaluate the effect of *B. paralicheniformis* T7 on the degradation process, hydrolysis was performed using an unfiltered enzymatic extract without sodium azide under the same conditions. Samples prepared under the same conditions but without enzymatic extracts were used as controls. After incubation, the samples were analyzed using scanning electron microscopy (SEM) to evaluate the degree of degradation. The samples were placed on stubs and sputtered with gold (10 nm). Images were captured using an Auriga CrossBeam 540 Scanning Electron Microscope (Carl Zeiss, Jena, Germany) at 3 kV.

### Whole-genome sequencing and mass spectrometric identification of extracellular proteases

The genome of *B. paralicheniformis* T7 was sequenced using the Oxford Nanopore method^31^ on a MinION sequencer (Oxford Nanopore Technologies Ltd., Oxford, UK). Genomic DNA libraries were constructed using an Oxford Nanopore Technologies Sequencing Kit (SQK-LSK109). This involved DNA fragmentation, followed by adapter ligation (Ligation Sequencing Kit, SQK-LSK109; Oxford Nanopore Technologies Ltd). The resulting libraries were quantified using Qubit 2.0 (Invitrogen, Carlsbad, CA, USA) and subjected to sequencing on a MinION platform with an FLO-MIN106D flow cell (R9; Oxford Nanopore Technologies Ltd.). The annotated genome was deposited in the NCBI GenBank. A library of amino acid sequences for all strains was generated based on the annotated genome of *B. paralicheniformis* T7. The enzymatic extract was concentrated 150-fold on a Pierce™ Protein Concentrator (10 K MWCO: 10 kDa molecular weight cutoff; Thermo Fisher Scientific) for mass spectrometric analysis of the secretory proteome. Protein separation was performed using 12% SDS-PAGE. Proteins were extracted from the gel and treated with trypsin (Promega) to form peptides. Subsequently, the peptides were separated on an Acclaim Pep-Map RSLC column (Thermo Fisher Scientific) using an acetonitrile gradient. An unmodified CaptiveSpray ion source was used to interface the HPLC system with a Maxis Impact II Instrument (Bruker Daltonics). The mass range of the MS scan was set to m/z 150–2,200 in positive ion polarity mode. *B. paralicheniformis* T7 extracellular proteases were identified on the Mascot platform using an amino acid sequence library of the strain.

### Fermentation of *B. Paralicheniformis* T7 in a bioreactor and enzyme preparation

A 10-L Biostat Bioreactor (Sartorius, Göttingen, Germany) was used to test the ability of *B. paralicheniformis* T7 to produce proteolytic enzymes during submerged fermentation. Submerged fermentation was performed according to the^33^. A single colony was inoculated into 5 mL of nutrient broth and cultured at 37 °C in a shaker incubator at 180 rpm for 18 h. Afterward, the culture was transferred to 200 mL of feather medium supplemented with 0.2% yeast extract and grown for 24 h under the same conditions. In a 10-L fermenter, the culture was inoculated into 6 L of sterile feather medium supplemented with 0.2% yeast extract. The conditions for submerged fermentation were as follows: temperature, 37 °C; agitation, 450 rpm; aeration, 6–10 L/min; and culturing time, 48 h. Samples were taken periodically to determine the number of colony-forming units and to measure protease activity.

The culture was cleared of cells and substrate residues by centrifugation at 11,000 × *g* and sterilized by microfiltration on a UPIRO-018 bench filtration apparatus (Vladisart, Vladimir, Russia) using an MKM46020 membrane polyethersulfone module (Vladisart) with a cutoff threshold of 0.22 μm. Next, 150 mL of sterile culture was concentrated by evaporation for 1 h on an RV05 basic rotary evaporator (IKA-Werke, Staufen, Germany) at 55 °C and 50 rpm under vacuum (4.9 kPa) until a volume of 3 mL was reached. Subsequently, 450 mL of sterile culture was frozen at -80 °С in a U570 Ultra-low freezer (New Brunswick Scientific, Enfield, CT, USA) and dried in a BETA 2–8 LDplus (Christ, Osterode, Germany) at -90 °С under vacuum (0.028 mBar) for 48 h in accordance with^34^. The lyophilized enzyme was ground into a powdered state, and the remaining sterile culture was spray-dried on an OM-1500 A spray dryer (Shanghai Pilotech Instrument & Equipment Co., Ltd., Shanghai, China) under the following conditions: chamber inlet temperature, 55 °C; chamber outlet temperature, 40 °C; spray pulse duration, 1 s; extract delivery rate, 4.5 mL/min.

### Software tools, bioinformatics, and statistical analysis

The chromatograms were analyzed after capillary sequencing using the Vector NTI Advance 11 program (Thermo Fisher Scientific). After nanopore sequencing, the sequences were annotated using Prokka Rapid Prokaryotic Genome Annotation Software (Prokka) version 1.13.14 (https://github.com/tseemann/prokka)^35^ and DNA Features Viewer software version 3.1.3 (https://github.com/Edinburgh-Genome-Foundry/DnaFeaturesViewer) to rapidly annotate the prokaryote genome. The Mascot platform was used for mass spectrometric proteins. The online resource Peptide Signal IP 5.0 (http://www.cbs.dtu.dk/services/SignalP/) was used for signal peptide prediction. All activity experiments were performed in triplicate. Enzymatic activity measurement data were obtained through independent activity assays, with mean values, standard deviations (SD), and p-values calculated using GraphPad Prism version 8.0.1 (GraphPad Software, La Jolla, CA, USA, www.graphpad.com). All data are presented as the mean ± SD (*n* = 3). Statistical significance between two groups was determined using the Student’s t-test, with a significance threshold of *P* < 0.05.

## Results

### Isolation and identification of proteolytic strain *B. paralicheniformis* T7

**Fig. 1:**
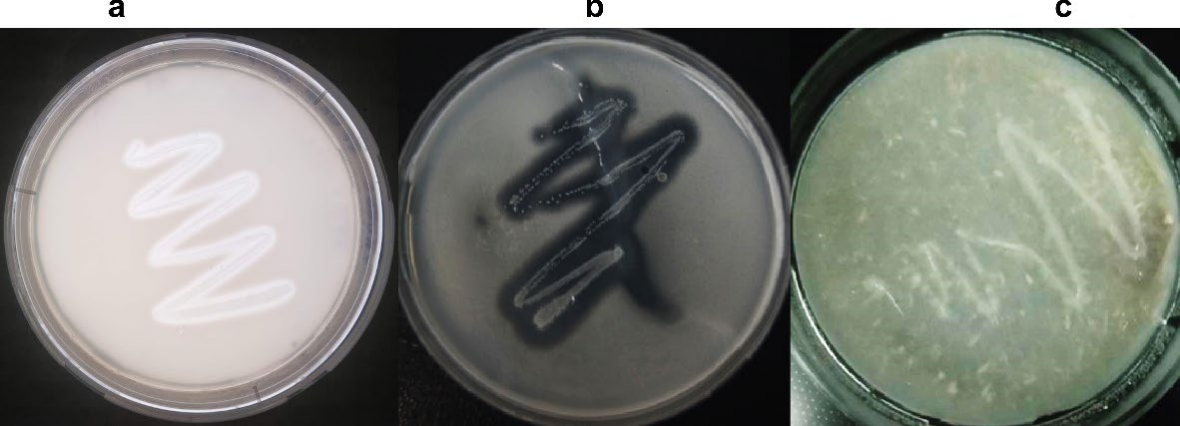
Т7 strain on skim milk (**a**), gelatin (**b**) and feather (**c**) agar after 24 h incubation at 37 °C.

A strain with proteolytic activity was isolated from soil samples collected near Taraz City, Kazakhstan (42.9 N 71.36667 E). After testing the seven isolates for proteolytic activity, T7 was found to have the highest proteolytic and keratinolytic activity (Fig. 1).

Taxonomic and morphological analyses of the colonies revealed that isolate T7 formed irregularly shaped colonies with turbid and opaque areas. These colonies were milky in color, irregularly shaped, 2–4 mm in diameter, and had glossy surfaces. When the T7 isolate was cultured in nutrient broth for 24 h, the strain exhibited average growth. During the growth process, the medium became turbid, and bacterial flakes formed in the culture medium. A hard-to-break film was formed at the liquid–air interface.

Gram staining of the strain revealed the presence of gram-positive straight rod-shaped bacteria, characteristic of bacteria belonging to the genus *Bacillus*. Bacterial cells were found alone or in chains. Schaeffer–Fulton staining demonstrated the ability to produce subterminal spores. Sanger sequencing of 16 S rRNA and complete genome sequencing using the Oxford Nanopore method revealed that strain T7 belonged to the species *B. paralicheniformis*.

### The effect of pH and temperature on the enzymatic activity of the extract

**Fig. 2:**
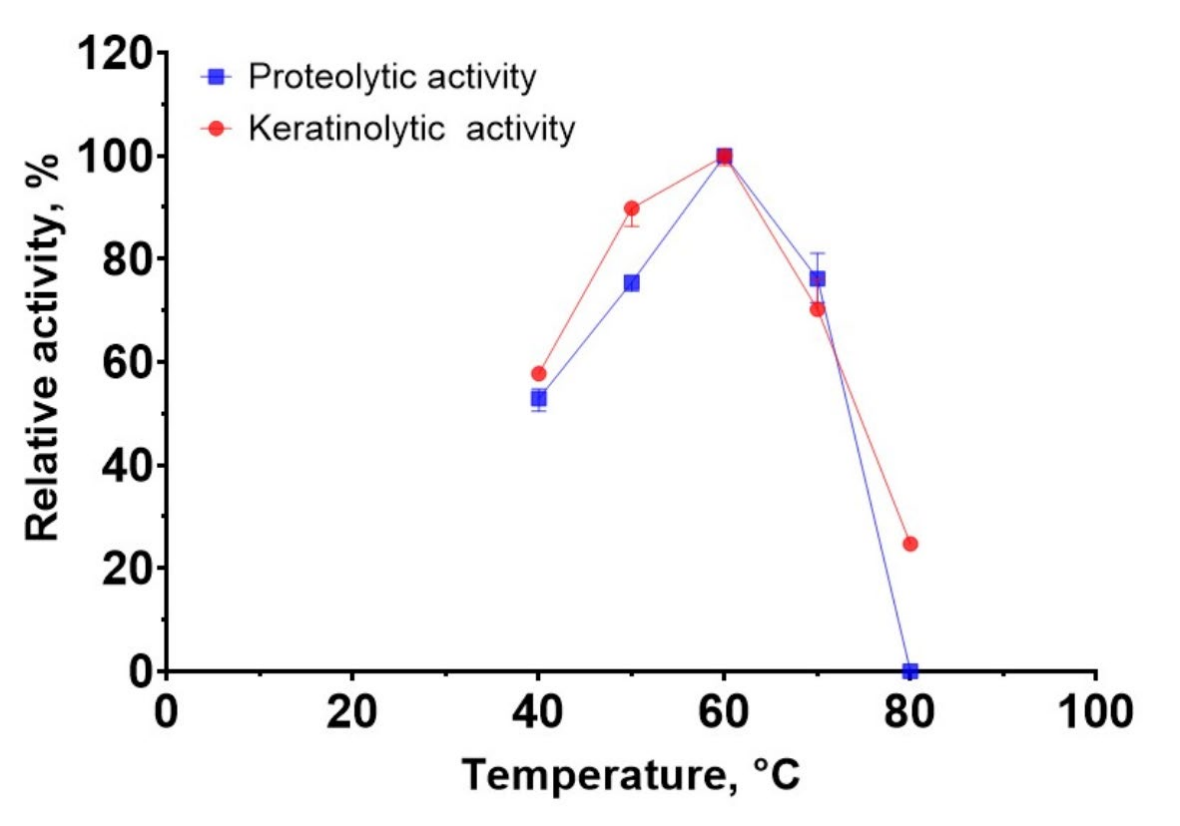
Effect of temperature on proteolytic and keratinolytic activity of *Bacillus paralicheniformis* T7 enzymatic extract.

The dependence of the activity of the enzymatic extract of *B. paralicheniformis* T7 on temperature at 30–80 °C was studied using azocasein and azokeratin. The extract showed maximum activity at 60 °C on both substrates (Fig. 2). The proteolytic enzymes in the extract retained 60% activity at 40–70 °C.

**Fig. 3:**
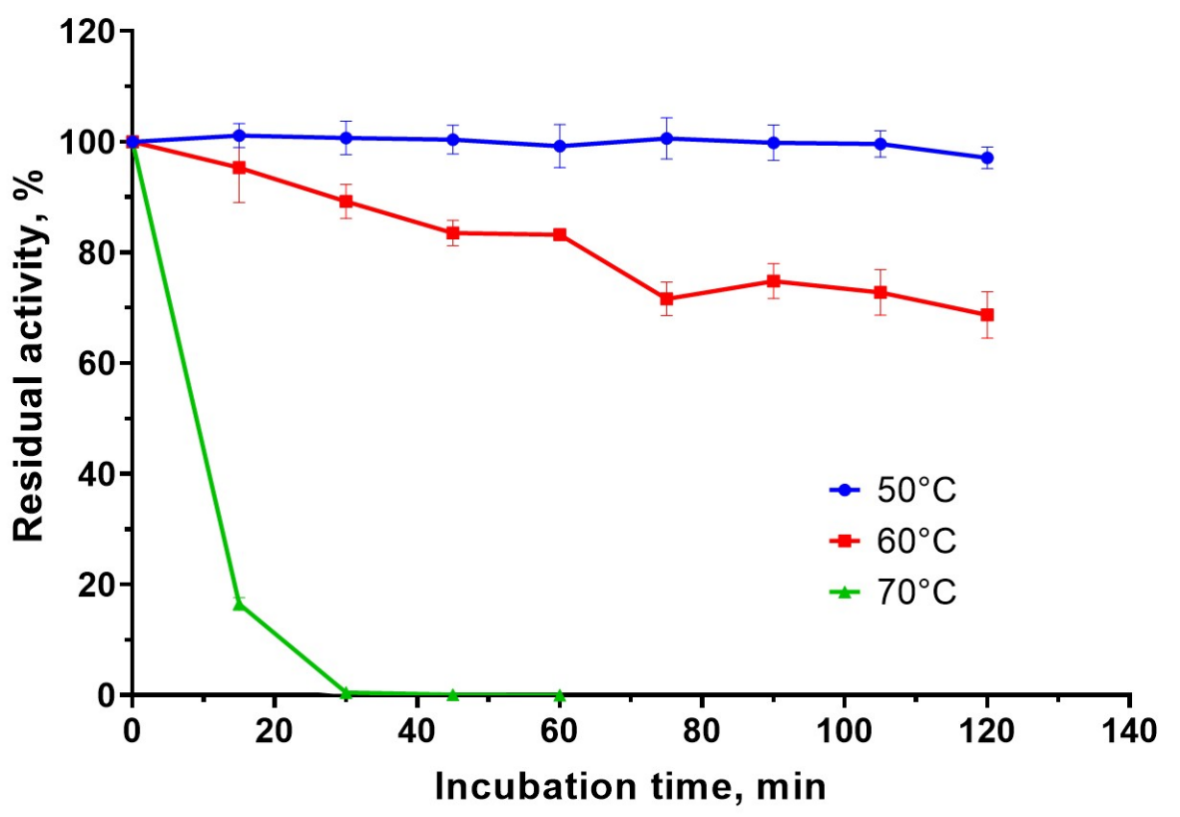
Residual proteolytic activity of *Bacillus paralicheniformis* T7 enzymatic extract after 2 h incubation at 50 °С, 60 °С, and 70 °С.

Figure 3 shows the residual proteolytic activity of the enzymatic extract after incubation for 2 h at 50, 60, and 70 °C. The extract retained 100% activity throughout incubation at 50 °C; after incubation at 60 °C, the residual activity was 83%. When incubated at 70 °C, the residual activity after 15 min was 16.5%; after 30 min, it was less than 0.5%.

**Fig. 4:**
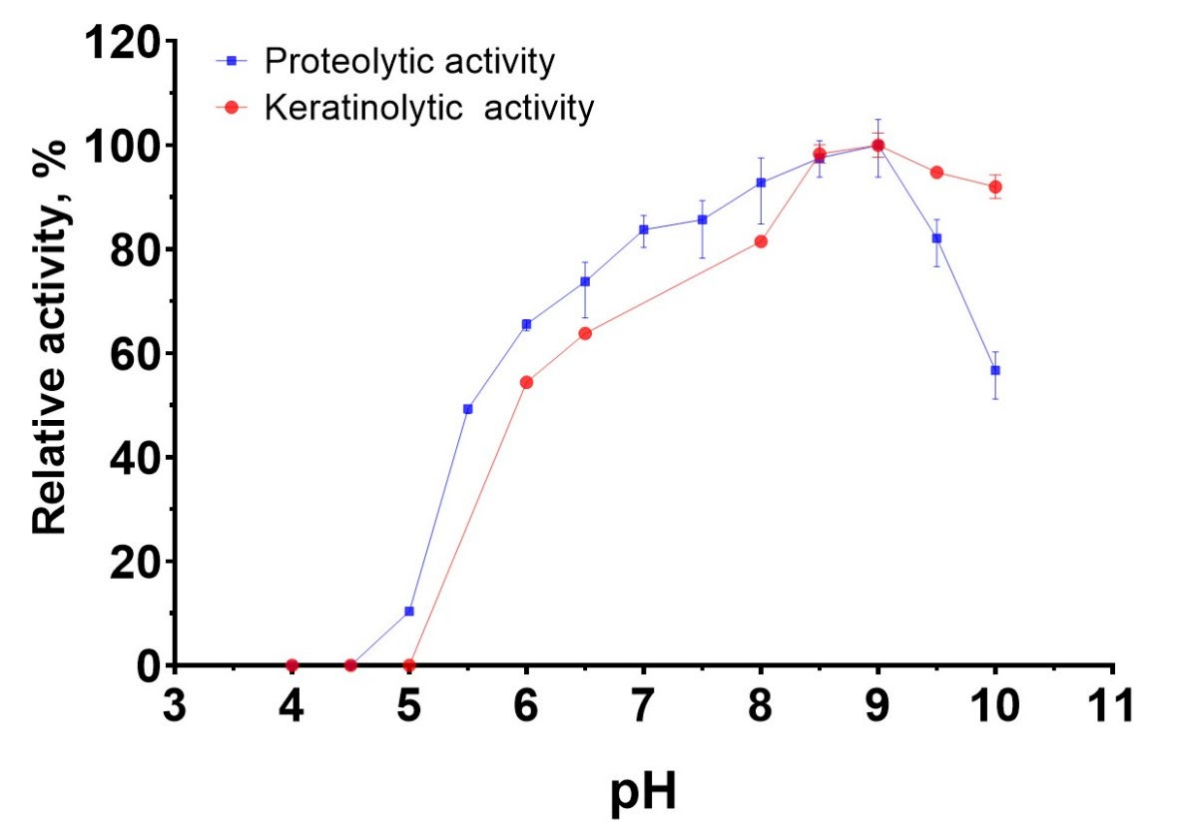
Effects pH on proteolytic and keratinolytic activity *Bacillus paralicheniformis* Т7 enzymatic extract.

The pH dependence of the proteolytic activity of the enzymatic extract of *B. paralicheniformis* T7 against azocasein and azokeratin was studied in the pH ranges of 6–11 and 4–10, respectively. The results showed that the proteolytic enzymes were active over a wide pH range and retained more than 60% of their activity at pH 6.0–10.0. The optimum conditions for all activities were achieved in Tris-HCl buffer with a pH of 9.0 (Fig. 4).

### Effect of metal ions, detergents, reducing agents, and inhibitors on enzymatic stability

A study on the effect of metal ions on the activity of the proteolytic enzymes of *B. paralicheniformis* T7 showed that Na⁺ and K⁺ did not affect the proteolytic and keratinolytic activities of the enzymatic extract (Table 1). Ca^2^⁺ at a concentration of 10 mM reduced proteolytic and keratinolytic activities by 25 and 12%, respectively. At concentrations of 5 and 10 mM, Ni^2+^ reduced proteolytic activity by 19 and 57%, respectively, but did not inhibit caseinolytic activity. Mg^2+^ did not affect either proteolytic or keratinolytic activity. At a concentration of 10 mM, Mn^2+^ reduced proteolytic activity by 69%. Cu^2+^ at concentrations of 5 and 10 mM reduced proteolytic activity by 24% and increased keratinolytic activity by 21 and 55%, respectively. The addition of Co^2+^ reduced the proteolytic activity of the extract by 60–30% and increased keratinolytic activity 2.7–3.7-fold. Cd^2+^ inhibited both proteolytic (50–78%) and keratinolytic activity (up to 40%). No pronounced effect was observed for Ba^2+^ ions, whereas Fe^3+^ ions were strong inhibitors of proteolytic activity.

**Table 1:** Effects of metal ions on proteolytic and keratinolytic activity of *Bacillus paralicheniformis* T7 enzymatic extract.

Metal ion	Residual proteolytic activity (%)		Residual keratinolytic activity (%)	
	Metal ion concentration 5 mM	Metal ion concentration 10 mM	Metal ion concentration 5 mM	Metal ion concentration 10 mM
Control	100 ± 1.0	100 ± 2.50	100 ± 0.31	100 ± 0.29
Na^1+^	102.23 ± 0.69	103.49 ± 3.56	106.13 ± 3.37	93.72 ± 5.32
K^1+^	100.64 ± 0.07	100.46 ± 2.75	111.21 ± 10.22	94.48 ± 2.99
Ca^2+^	92.82 ± 0.035	75.27 ± 3.98	106.52 ± 7.69	87.97 ± 1.73
Ni^2+^	81.77 ± 1.12	43.30 ± 0.67	128.53 ± 8.66	99.4 ± 4.78
Mg^2+^	100.23 ± 1.32	101.90 ± 3.82	100.69 ± 12.67	93.12 ± 4.12
Mn^2+^	98.13 ± 2.88	30.98 ± 1.46	135.09 ± 3.32	92.97 ± 7.15
Cu^2+^	76.96 ± 1.55	76.59 ± 1.87	121.04 ± 5.03	45.84 ± 2.61
Co^2+^	108.37 ± 2.36	87.16 ± 6.29	52.71 ± 7.68	40.91 ± 1.11
Cd^2+^	50.62 ± 1.87	22.55 ± 2.40	88.66 ± 5.56	59.08 ± 3.63
Ba^2+^	97.05 ± 1.17	85.63 ± 0.90	108.03 ± 7.56	90.93 ± 2.83
Fe^3+^	23.21 ± 0.5	0	25.30 ± 3.49	5.32 ± 1.28

Protease inhibitors, detergents, and reducing agents were tested for their effects on the keratinolytic and proteolytic activities of the enzymatic extract. The results showed that β-mercaptoethanol, at concentrations of 5 and 10 mM, increased the yield of azokeratin hydrolysis products but did not affect the enzyme’s ability to break down azocasein (Table 2). In addition to increasing the production of azokeratin hydrolysis products, 5 and 10 mM DTT decreased the proteolytic activity of the enzymatic extract by 19 and 28%, respectively.

**Table 2:** Effects of reducing agents, ionic and nonionic detergents, protease inhibitors on the activity of *Bacillus paralicheniformis* T7 enzymatic extract.

Reagent	Residual proteolytic activity (%)	Residual keratinolytic activity (%)	*P* value (proteolytic activity)	*P* value (keratinolytic activity)
Control	100 ± 2.31	100 ± 0.21	-	-
β-mercaptoethanol 5 mM	102.69 ± 3.92	117.08 ± 5.3	0.2207	0.0158
β-mercaptoethanol 10 mM	103.58 ± 2.80	110.45 ± 2.09	0.1283	0.038
Dithiothreitol 5 mM	81.30 ± 2.56	137.07 ± 7.27	0.0004	0.0001
Dithiothreitol 10 mM	72.74 ± 2.55	140.40 ± 4.04	0.0001	0.002
Tween 20 1%	87.67 ± 5.04	69.75 ± 5.89	0.0416	0.004
Tween 20 5%	78.39 ± 2.68	68.53 ± 10.99	0.0234	0.0223
Triton X-100 1%	79.38 ± 4.26	116.29 ± 7.95	0.0004	0.0917
Triton X-100 5%	76.84 ± 4.51	80.93 ± 5.37	0.0006	0.0354
Sodium dodecyl sulphate 1%	24.54 ± 3.67	13.07 ± 3.63	0.0001	0.00001
PMSF 5 mM	11.76 ± 3.28	10.26 ± 8.13	0.00002	0.0005
PMSF 10 mM	0	0	0.000002	0.00001
EDTA 5 mM	39.79 ± 0.61	78.42 ± 3.12	0.00001	0.0005
EDTA 10 mM	41.51 ± 0.64	78.4 ± 3.41	0.00003	0.0012
Pepstatin A 35 mM	90.58 ± 3.69	101.94 ± 1.68	0.079	0.6087
Pepstatin A 50 mM	88.87 ± 6.29	109.25 ± 8.53	0.09643	0.301
E64 35 μm	83.41 ± 0.96	83.33 ± 2.02	0.0028	0.0004

Tween 20 reduced the proteolytic and keratinolytic activities of the extract by 13–30%. Triton X-100 reduced proteolytic activity by 20%. The addition of 1% Triton X-100 increased keratinolytic activity by 16%, and 5% Triton X-100 reduced keratinolytic activity by 20%. SDS is a strong inhibitor of keratinolytic activity; after adding 1% SDS, the residual activity of the enzymatic extract was 13%.

The addition of protease inhibitors to the enzymatic extract revealed that 5 mM PMSF reduced both the proteolytic and keratinolytic activities by 90%, whereas increasing the PMSF concentration to 10 mM completely inhibited both extract activities. EDTA also had an inhibitory effect, reducing proteolytic and keratinolytic activity by 60 and 22%, respectively. Pepstatin A slightly reduced proteolytic activity but did not affect the keratinolytic activity of the extract. E64 also showed a minor inhibitory activity on the proteolytic activity of the extract.

### Substrate specificity

**Fig. 5:**
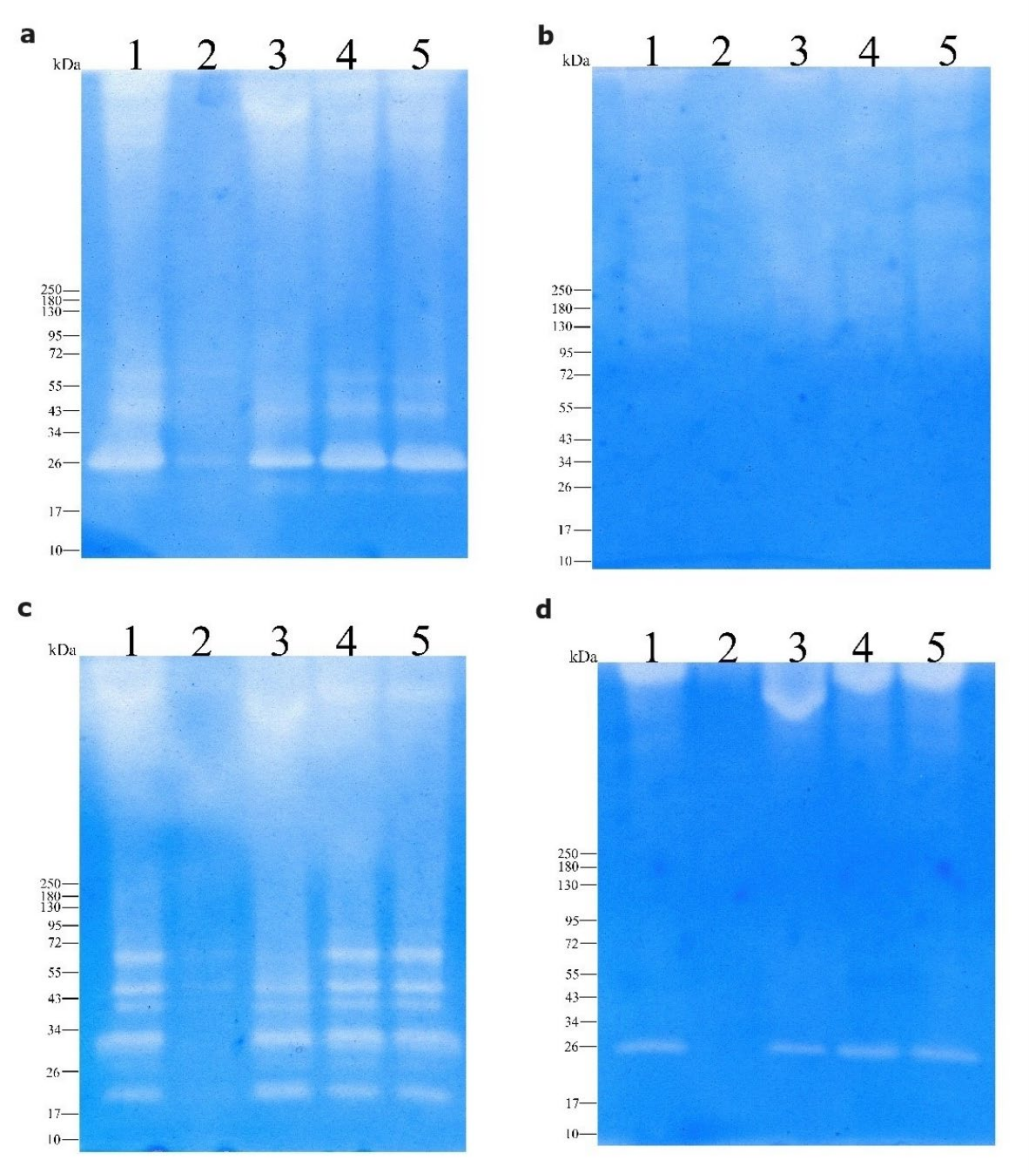
Zymogram with copolymerized casein (**a**), keratin (**b**), gelatin (**c**), and BSA (**d**) for the enzyme extract of *B. paralicheniformis* T7. Enzymatic extract (lane 1), enzymatic extract with PMSF (lane 2), enzymatic extract with EDTA (lane 3), enzymatic extract with Pepstatin A (lane 4), and enzymatic extract with E64 (lane 5). The raw zymograms are presented in the supplementary materials.

Zymographic analysis revealed that the enzymatic extract of *B. paralicheniformis* T7 contained proteases that hydrolyzed casein, keratin, gelatin, and BSA (Fig. 5). PMSF inhibited the activity of the enzymatic extract of *B. paralicheniformis* T7 in reactions with all substrates, indicating that most proteases in the enzymatic extract belonged to the serine protease class. In gelatin zymography, enzymes with masses of 32, 40, and 45 kDa were inhibited by EDTA, indicating that they belong to the class of metalloproteases.

### Hydrolysis of hemoglobin, gelatin, keratin, casein, BSA, and ovalbumin

**Fig. 6:**
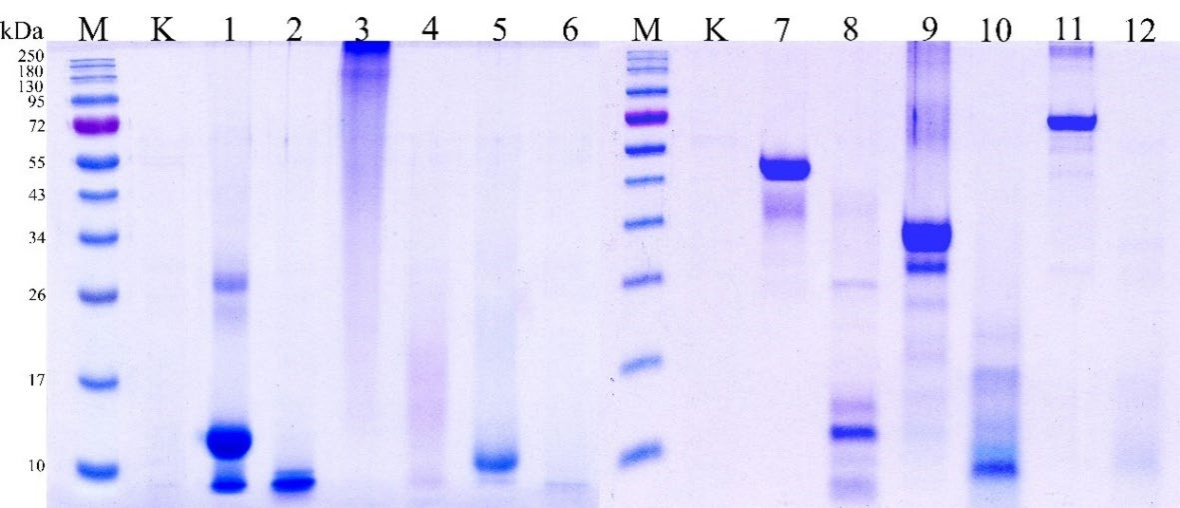
Hydrolysis of the proteins by enzyme extract of *Bacillus paralicheniformis* T7: М – protein marker NEB cat.#P7719S; K – enzymatic extract; 1 – hemoglobin, 2 – hydrolyzed hemoglobin in 5 min, 3 – gelatin, 4 – hydrolyzed gelatin in 5 min, 5 – keratin, 6 – hydrolyzed keratin in 30 min, 7 – ovalbumin, 8 – hydrolyzed ovalbumin in 1 min; 9 – casein; 10 – hydrolyzed casein in 15 s; 11 – BSA; 12 – hydrolyzed BSA in 5 min. The raw gels are presented in the supplementary materials.

The hydrolysis rate, which depends on the nature of the substrate, is critical for protein cleavage. To study the hydrolysis rate, the following proteins were selected: hemoglobin, gelatin, keratin, casein, BSA, and ovalbumin. All substrates were hydrolyzed at 60 °C in 0.1 M Tris-HCl buffer at pH 8.5. At the end of the experiment, electrophoretic separation of the hydrolysis products was performed by SDS-PAGE (Fig. 6).

The rate of hydrolysis of different substrates by the proteolytic enzymes in the enzymatic extract of *B. paralicheniformis* T7 differed remarkably. Casein was hydrolyzed faster than the other substrates (Fig. 6), being hydrolyzed after 15 s of treatment. One minute was sufficient to hydrolyze ovalbumin, while BSA, hemoglobin, and gelatin were hydrolyzed within 5 min. Feather keratin was most resistant to the hydrolytic action of *B. paralicheniformis* T7 enzymes; 1 mg of keratin was hydrolyzed in 30 min.

### Degradation of horn, hoof, hide, wool, and feather samples

**Fig. 7:**
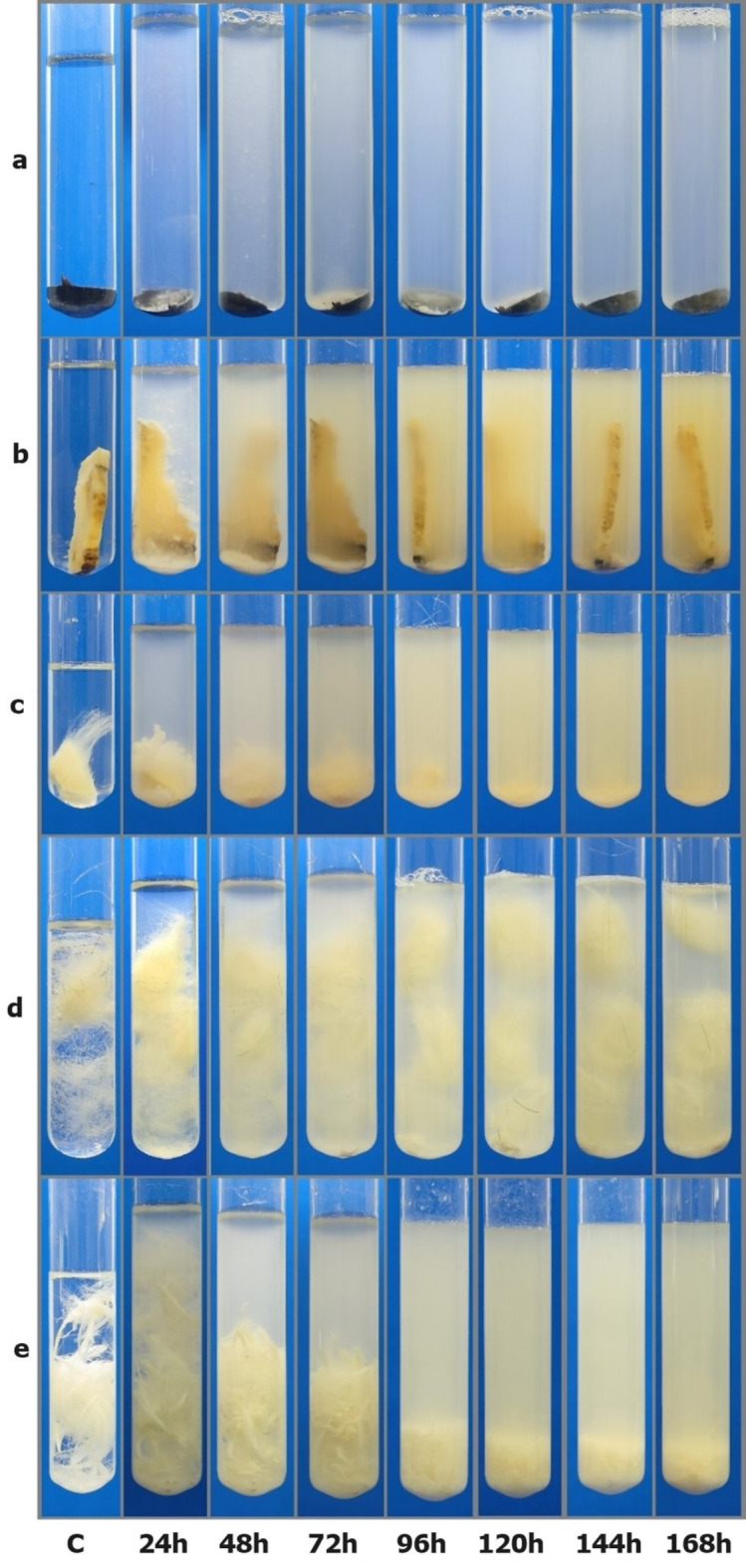
Degradation of horn (**a**), hoof (**b**), hide (**c**), wool (**d**) and feather (**e**) samples using *B. paralicheniformis* T7 culture for 168 h. C - control sample after 168 h of incubation without addition of bacterial culture.

Treatment of horn, hoof, hide, wool, and chicken feather samples with *B. paralicheniformis* T7 culture showed the degradative properties of the strain in all samples. Figure 7 shows the dynamics of horn, hoof, hide, wool, and feather sample degradation (Table 3).

**Table 3:** Information on the degradation of horn, hoof, hide, wool and feathers within 7 days.

Substrate	Hydrolysis degree (%)	Proteolytic activity (U/mL)	*P* value (proteolytic activity)	Keratinolytic activity (U/mL)	*P* value (keratinolytic activity)
horn	3.6	82.6 ± 3.27	0.56144	77.27 ± 1.76	< 0.0001
hoof	29.6	114.8 ± 2.12	0.00653	97.6 ± 3.05	< 0.0001
hide	97.2	53.4 ± 2.11	0.00175	25.93 ± 2.16	< 0.0001
wool	34.5	134.67 ± 1.36	0.12840	88.45 ± 8.63	0.75399
feather	100	134.4 ± 0.20	-	79.3 ± 5.28	-

The enzymes of the strain hydrolyzed the studied substrates differently, with the type of protein substrate used also affecting enzyme activity.

### Scanning electron microscopy

**Fig. 8:**
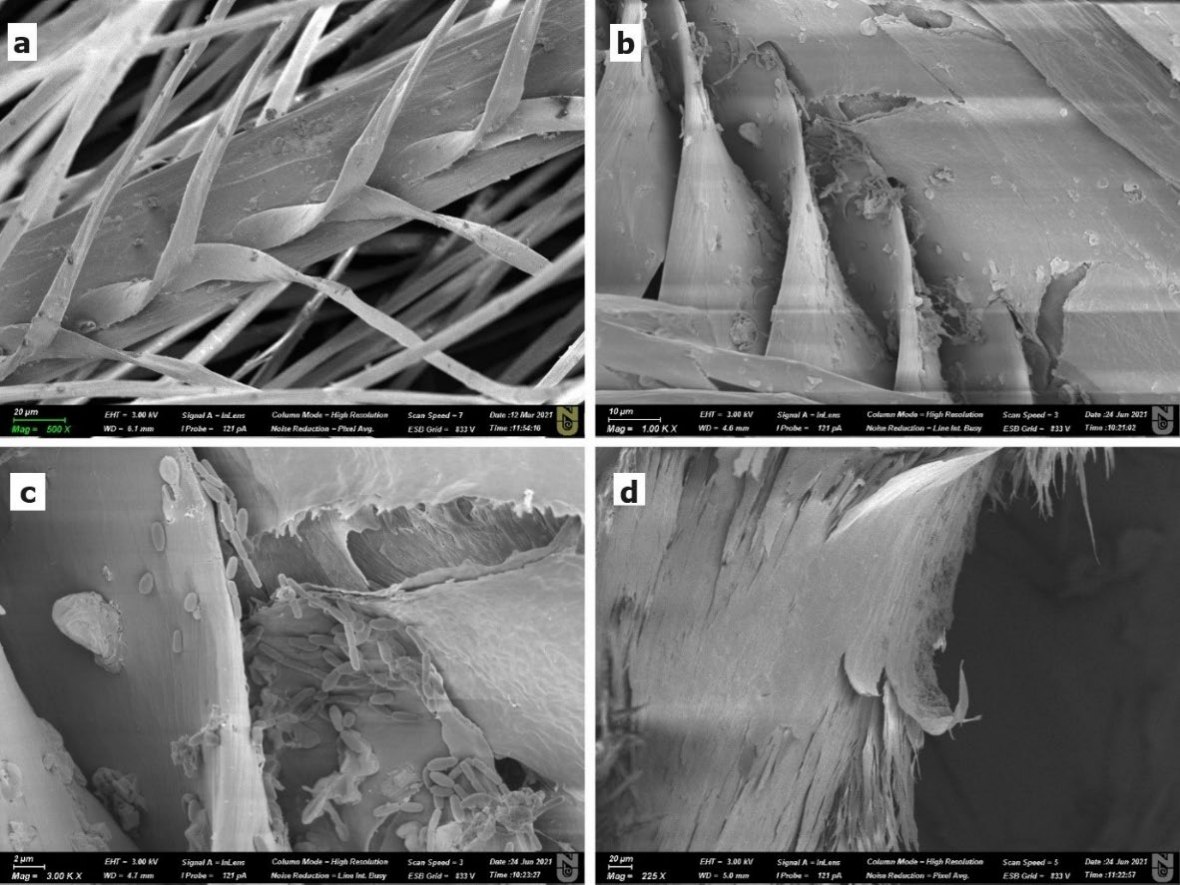
Scanning electron microscopy image of a chicken feather after hydrolysis by *B. licheniformis* T7 strain, where (**a**) is a whole feather, (**b**) and (**d**) is a feather after 48 h of incubation, (**c**) is a feather after 72 h of incubation. Scale bars in (**a**), (**d**): 20 μm; in (**b**): 10 μm and in (**c**): 2 μm.

Changes in the microstructure of chicken feathers during hydrolysis by *B. paralicheniformis* T7 enzymes were observed using scanning electron microscopy. Figure 8 shows images of feathers subjected to hydrolysis. Feather stems with beards and beards with hooks were clearly observed in the images of the untreated feathers (Fig. 8A). Damaged nicks were visible after 48 h of culture (Fig. 8B). The hooks and beards were almost completely decomposed after 72 h (Fig. 8C). *B. paralicheniformis* T7 cells clearly adhered to feather surfaces.

Besides enzyme action, the adhesion of live *B. paralicheniformis* T7 bacteria to feather substrates is important in feather destruction. Scanning electron microscopy enabled the visualization of bacterial cells and confirmed the direct involvement of *B. paralicheniformis* T7 bacteria in feather destruction (Fig. 8C).

The importance of live bacteria in feather hydrolysis was confirmed in feather destruction experiments with live bacteria, an enzymatic extract obtained by culture filtration through a 0.22-µm filter, and sodium azide addition. Feather destruction in samples containing live bacteria was faster than that in samples without microorganisms.

### Mass spectrometric and genomic analyses of the secretory proteome

The sequenced *B. paralicheniformis* T7 genome comprised 4,359,207 bp and 4,388 protein-coding sequences. In the closely related strain *B. paralicheniformis* MKU3, the genome has 4,370,039 bp encoding 4872 protein-coding genes^36^. The genome of *B. licheniformis* strain T5 also isolated from this soil sample has 4,247,430 bp encoding 5391 protein-coding genes^37^. The complete *B. paralicheniformis* T7 genome has been deposited in NCBI GenBank under accession number CP124861. Seven peptidases with molecular masses of 154.8, 86.2, 41.2, 53.3, 60.6, 30.5, and 26.8 kDa were identified in the enzymatic extract using HPLC-Q/TOF mass spectrometry and Mascot analysis against all *B. paralicheniformis* T7 proteins (Table 4).

**Table 4:** Proteases identified by protein mass spectrometry and proteomics in the enzymatic extract of the *Bacillus paralicheniformis* T7 strain.

Molecular weight, Da	Protein	DNA Locus	Protein length (AA)	Score
154,835	S8 family serine peptidase	2407118.2411416	1432	1777
86,179	S8 family serine peptidase	120102.122522	806	40
41,156	S8 family serine peptidase	2934598.2935733	378	1365
53,274	S41 family peptidase	1794133.1795523	463	27
60,574	M14 family metallocarboxypeptidase	3290537.3292180	547	256
30,548	M55 family metallopeptidase	2645411.2646235	274	308
26,825	M42 family metallopeptidase	2945002.2945730	242	1386

### Fermentation of *B. paralicheniformis* T7 in a bioreactor and production of proteolytic preparation

**Fig. 9:**
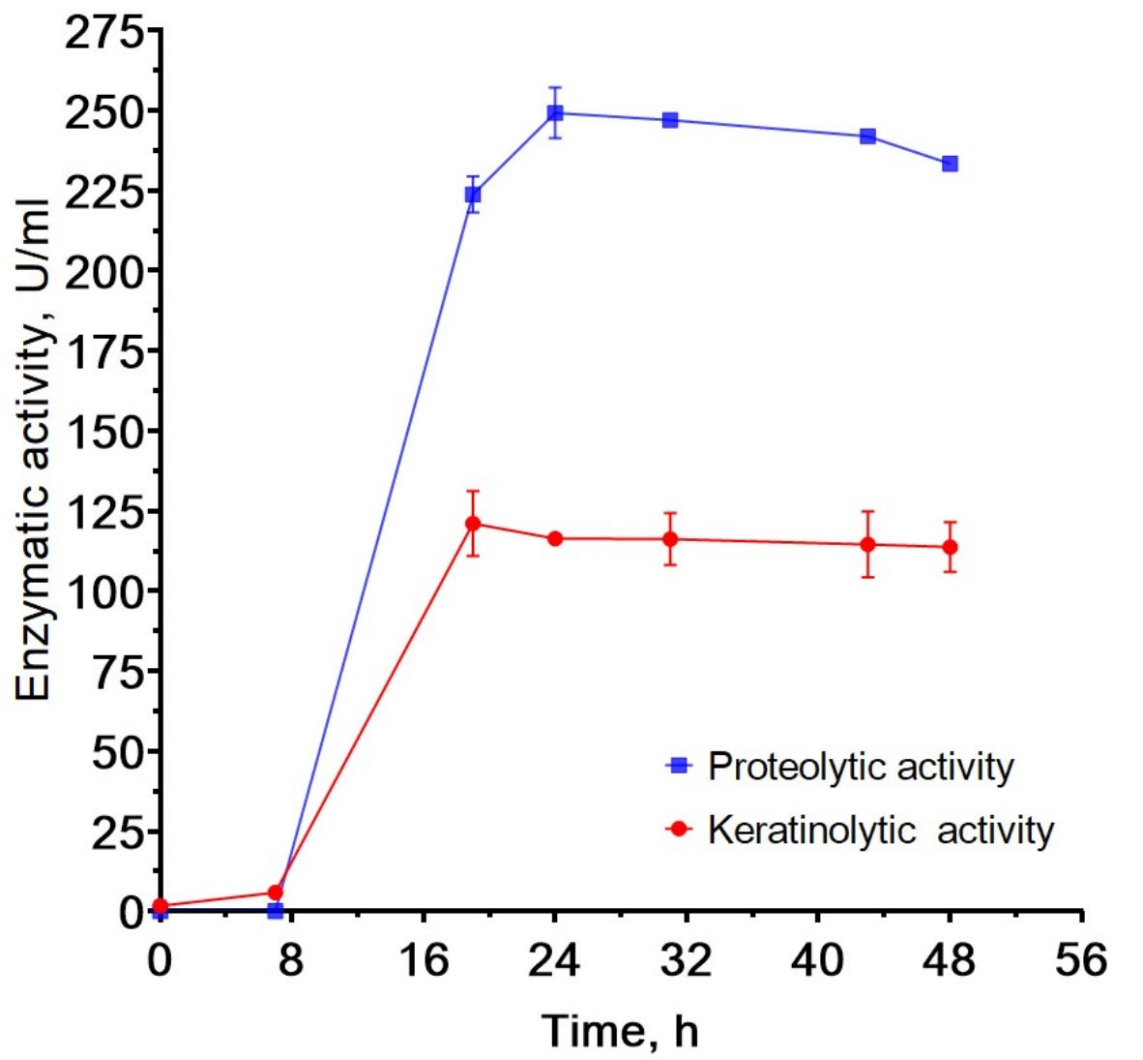
Pilot scale production of proteolytic enzymes by *B. paralicheniformis* T7.

To obtain proteolytic enzymes by the biotechnological method, *B. paralicheniformis* T7 was cultivated by submerged fermentation in a 10-L bioreactor. The density of the culture during fermentation was 0.3 × 10^9^, 1 × 10^9^, and 5.3 × 10^9^ CFU/mL after 18, 24, and 48 h of cultivation, respectively. Figure 9 shows the protease activity per unit volume as a function of fermentation time in the bioreactor. The results indicate that the maximum protease and keratinase activities were 249.20 ± 7.88 and 116.17 ± 8.10 U/mL, respectively.

The following methods were used to obtain enzyme preparations from the culture liquid after *B. paralicheniformis* T7 fermentation: concentration by vacuum evaporation at 55 °C for 1 h; lyophilic drying at -52 °C under vacuum for 63 h; spraying drying in an air stream at 55 °C. The enzymatic extract was concentrated by volume 50-fold by evaporation under negative pressure. The protease and keratinase activities of the obtained preparations were measured and presented in Table 5.

**Table 5:** Activity of the enzymatic extract after concentration, freeze-drying and spray-drying.

Form of enzyme extract	Activity	
	Protease activity	Keratinase activity
Concentrate enzyme extract	4 940 ± 30.0 U/mL	2 493 ± 1.42 U/mL
Lyophile powder	148.67 ± 6.89 U/mg	83.67 ± 1.04 U/mg
Spray Dried powder	193.67 ± 4.86 U/mg	83.17 ± 6.37 U/mg

## Discussion

Microbial proteases are in demand in various fields, including food and feed processing and detergent and pharmaceutical production. The cost of microbial enzymes is significantly lower than that of eukaryotic analogs because microorganisms produce enzymes faster than mammalian and plant cells. Furthermore, enzyme production does not depend on climatic conditions or seasonal changes and does not violate ethical or environmental standards^7^. Among proteases, keratinases (EC 3.4.21/24/99) can hydrolyze strong fibrillar proteins such as collagen and keratin, depending on their substrate specificity^38^. Keratinases efficiently cleave keratinous materials, such as feathers, nails, and wool, as well as soluble protein substrates, such as casein, whey proteins, and BSA^17^. A promising source of cheap proteinaceous raw materials is keratinous waste from animal husbandry, including feathers, horns, hooves, wool, and hides. For example, chicken feathers account for approximately 5–7% of the total weight of a chicken^34^, and hides, horns, and hooves account for 11% of cattle weight. The high proteolytic activity of keratinases enables them to hydrolyze highly resistant keratins, which is why they are considered the most promising proteolytic enzymes in the industry^39,40^.

*Bacillus* has an efficient enzymatic system, including various proteolytic enzymes that enable it to occupy different ecological niches^7^. *Bacillus* spp. are ubiquitous in various soil types, compost, and landfills. They are predominantly mesophilic microorganisms; however, several of their enzymes show increased thermostability^41,42^. The productive properties of *Bacillus* have allowed this taxon to occupy a dominant position in the microbial synthesis industry^43^. Among the keratin-degrading microorganisms, bacilli are known keratinase producers^38–42^, some of which have been isolated from soil and poultry waste^22,44^.

Strain T7 was isolated from Kazakhstani soil and identified as *B. paralicheniformis* based on culture, morphological, proteomic, molecular genetic, and genomic characteristics. Studies have shown that when cultured on milk, keratin, and gelatin agar, this strain produces proteolytic enzymes that hydrolyze the corresponding substrates, forming characteristic clear zones. *B. paralicheniformis* T7 can grow on feather medium with added salts, in which feather keratin is the only source of organic matter.

Studies on the biochemical characteristics of the enzymatic extract of *B. paralicheniformis* T7 indicated that the proteolytic enzymes it produces have an optimum pH of 9.0. Compared with the described proteases, most bacillary proteases and keratinases are alkaline (Table 6), except for proteases from *B. velezensis* Y1^45^, *Bacillus* sp. ZG20^5^, and keratinases from *B. licheniformis* dcs1^46^. *B. paralicheniformis* T7 proteases and keratinases display maximum activity at 60 °C. Table 6 shows that, among bacillary proteases and keratinases, many enzymes have similar temperature characteristics^22,47–54^.

**Table 6:** Temperature and pH optimum of various bacillary proteases and keratinases.

Strain	Optimal temperature (°C)	pH optimum	Reference
Proteases			
* B. paralicheniformis* T7	60	9.0	This work
* Bacillus* sp. A5.3	70	10.5	^22^
* B. altitudinis* W3	45–55	8.5–10.5	^55^
* Bacillus* sp. RAM	50	9.0	^56^
* B. licheniformis* K7A	70	10.0	^57^
* B. velezensis* Y1	50	6.0	^45^
* B. safensis* RH12	60	9.0	^47^
* Bacillus* sp. DPUA 1728	50	9.0	^58^
* Bacillus* sp. ZG20	45	7.0	^5^
* Bacillus* sp. CL18	55	8.0	^59^
* B. clausii* GMBE 22	60	12.0	^60^
* B. amyloliquefaciens* D1	50	6.0	^61^
Keratinases			
* B. paralicheniformis* T7	60	9.0	This work
* Bacillus* sp. A5.3	60	8.5	^22^
* B. licheniformis* PWD-1	50	7.5	^32^
* B. subtilis* KD-N2	55	8.5	^62^
* B. licheniformis* RPk	60	9.0	^48^
* B. subtilis* MA21	60	9.0	^49^
* B. zhangzhouensis* BK111	60	9.5	^50^
* B. cereus* YQ15	60	10.0	^52^
* B. subtilis* SCK6	60	10.0	^53^
* B. licheniformis* dcs1	45	7.0	^46^
* Bacillus* sp. Nnolim-K1	60	8.0	^54^
* Bacillus* sp. BK111	60	9.5	^50^
* B. paralicheniformis* FUM-2	45	11	^24^

The thermostability study revealed that the proteolytic enzymes in the enzymatic extract exhibited enhanced thermostability. The proteases retained activity after a 2-h preincubation at 50 °C, and residual activity was lowered by 17% after preincubation at 60 °C. Following a preincubation period of 70 °C, the proteases exhibited reduced stability. In this instance, 83.5% of extract protease activity was lost after 15 min, and less than 0.5% remained after 30 min. Nevertheless, the thermostability of *B. paralicheniformis* T7 enzymes was greater than those of *Bacillus* sp. A.5.3^22^. Additionally, *B. paralicheniformis* T7 proteases are more thermostable than those of *B. velezensis* Y1, which lose over 80% of their activity following preincubation for 2 h at 55 °C and 10 min at 60 °C^45^.

Single-charged ions were shown not to affect the proteolytic and keratinolytic activities of the enzymatic extract of *B. paralicheniformis* T7. Among doubly charged ions, Ca^2^⁺, Mg^2^⁺, and Ba^2^⁺ have relatively neutral effects. Ni^2^⁺, Mn^2^⁺, and Cd^2^⁺ at a concentration of 10 mM strongly inhibited proteolytic activity. At a concentration of 10 mM, Cu^2^⁺ and Cd^2^⁺ negatively affected keratinolytic activity. Co^2⁺^ at a concentration of 5 mM did not reduce proteolytic activity, only at a concentration of 10 mM did it slightly reduce proteolytic activity by 13%. At the same time, a decrease in keratinolytic activity by 59 − 48% was observed at both 5 mM and 10 mM Co^2⁺^. Fe^3⁺^ had the greatest inhibitory effect; the addition of 5 mM residual protease and keratinase activity was 23% and 25%, respectively. And 10 mM FeCl_3_ completely inactivated proteolytic activity and reduced keratinolytic activity by 95%. This sensitivity to metal ions is typical for metalloproteases. Therefore, keratinases in the extract had a higher tolerance to metal ions than that of proteases. The increase in the yield of keratin hydrolysis products following the addition of reducing agents can be explained by their reducing effect on the disulfide bonds between cysteine residues within keratin^14^. β-mercaptoethanol at 5 and 10 mM concentrations has no significant effect on proteolytic activity (*P* > 0.05), but causes a slight increase in keratinolytic activity, with significant P values (0.0158 and 0.038). In contrast, dithiothreitol (DTT) at 5 and 10 mM significantly decreases proteolytic activity (*P* < 0.001) but increases keratinolytic activity (*P* < 0.01). Ionic detergents such as Tween 20 and Triton X-100 lead to a decrease in proteolytic activity, as evidenced by significant P values (0.0416 for Tween 20 and 0.0004 for Triton X-100). However, their effect on keratinolytic activity varies: Twin 20 reduces activity with a high level of significance (*P* < 0.05), while Triton X-100 at a concentration of 1% causes no significant change (*P* = 0.0917). Among the protease inhibitors, the strongest inhibition was observed in the presence of PMSF and EDTA, where enzyme activity was significantly inhibited at both concentrations (*P* < 0.001). These data confirm that the enzyme systems are sensitive to these inhibitors. Qualitative and quantitative experiments on the effects of the protease inhibitors PMSF, EDTA, pepstatin A, and E64 showed that the proteolytic and keratinolytic activities of the enzymatic extract were primarily attributed to serine proteases. Metalloproteases also participate in protein hydrolysis but contribute less to the total activity, which is probably due to their low level of expression and possibly low specific activity. The action of asparagine and cysteine proteases is negligible. The inhibitors Pepstatin A and E64 have less pronounced effects; the P value indicates insignificant changes in activity. The proteolytic activity of the enzymatic extracts of *Bacillus* sp. A5.3 and other *Bacillus* representatives has been shown to be strongly inhibited by PMSF^44,63–67^, and the negative effects of EDTA on proteolytic enzyme activity have also been reported for proteases from *B. halodurans* PPKS-2^67^, *Bacillus* sp. MKR5^68^, and *Chryseobacterium* sp. kr6^69^. Zymographic analysis of casein, keratin, gelatin, and BSA belonging to different protein structural classes revealed their hydrolysis by proteases of different molecular masses. Similar zymographic data were obtained for the extracts of *B. subtilis* SCL, *B. subtilis* 1271, and *Bacillus* sp. A5.3^22,66,70^. The zymograms show that BSA is hydrolyzed by one serine protease with an estimated mass of 26 kDa, which is also visible in the zymogram with casein, along with two other proteases. Five proteases were involved in gelatin hydrolysis. Protein hydrolysis showed that milk casein was the most susceptible to hydrolysis; 1 mg was completely hydrolyzed in 15 s. Ovalbumin was the second most susceptible to hydrolase action, with 1 mg being hydrolyzed in 1 min. BSA, hemoglobin, and gelatin were more resistant to the action of *B. paralicheniformis* T7 proteases, requiring 5 min for hydrolysis. *B. paralicheniformis* T7 keratinases hydrolyzed 1 mg of keratin in 30 min. Compared with that of *Bacillus* sp. A5.3, *B. paralicheniformis* strain T7 extract is comparable in terms of keratinolytic activity but superior in proteolytic activity. *B. paralicheniformis* T7 enzymes required much less time to hydrolyze casein, ovalbumin, and BSA than proteases from *Bacillus* sp. A5.3^22^.

The presence of several clear zones in all the zymograms indicated that the enzymatic extract was a cocktail of proteases of different molecular masses in range 20–200 kDa. A similar zymogram with many bands has been reported for *B. subtilis* SCL, *B. subtilis* 1271, *B. paralicheniformis* MKU3^36,70,71^. *B. paralicheniformis* T7 enzymes are characterized by high proteolytic activity against keratin- and collagen-containing substrates. The proteases of this strain can hydrolyze substrates such as wool, hide, feathers, horns, and hooves, which are resistant to the action of most proteases. β-keratin, a component of feathers, and α-keratin (horns, hooves, and wool) have high mechanical strength and resistance to the action of most proteases^72^, which requires the synergistic action of enzymes^12^.

Comparative analysis revealed that the horn was the most resistant to protease action; more than 96% of horns remained unhydrolyzed after 7 days of co-culture with *B. paralicheniformis* T7 cells. Chicken feathers and cattle hides were the least resistant to hydrolysis. Hooklet and barbule degradation was observed on the feathers after 2 days. Barb degradation began on the third day, and feathers were completely hydrolyzed by the end of the fourth day. The dermis of the hide was also highly sensitive to *B. paralicheniformis* T7 proteases, and at the end of incubation, only wool remnants were observed in the sample, with the culture losing transparency and acquiring a milky color. The P value for proteolytic activity indicated statistically significant differences for hoof, hide, and horn, while the values for wool and feathers were less significant. For most substrates except wool, the P value for keratinolytic activity was significant. The resistance of the keratinous substrates to the proteolytic action of *B. paralicheniformis* T7 was in the order of horn > hoof > wool > hide > feather. *B. paralicheniformis* T7 degrades feathers 120 h faster than does *Bacillus* sp. A5.3^22^. The keratinous substrate hydrolysis results indicated that feathers and hides are promising inexpensive substrates for producing protein hydrolysates.

A comparative analysis of the protease and keratinase activities of *B. paralicheniformis* T7 cultured on different substrates revealed that wool and feathers effectively induced protease activity, whereas hooves and wool stimulated keratinase activity. The lowest values for both activities were observed when hides were used. These findings indicate that wool and feathers are excellent stimulators of proteolytic and keratinolytic enzyme production, respectively. Previously, the determining effect of chicken feathers as a substrate on the production of keratinases of *B. paralicheniformis* strain MKU3 was also noted^25^. In contrast to *B. paralicheniformis* MKU3, which showed no collagenase activity, this activity was observed for *B. paralicheniformis* strain T7, as confirmed by a zymographic test on gelatin and more than 97% skin hydrolysis.

The SEM visual analysis of hydrolyzed chicken feathers revealed that their hydrolysis was much more efficient using bacterial culture than using enzymatic extract alone. The SEM results showed that *B. paralicheniformis* T7 cells tightly adhered to the feather surface, intensifying feather degradation at the attachment sites, consistent with previous results^12,22,70^. Without live bacterial cells, the extracellular keratinolytic activity of *Bacillus* sp. A5.3 and *B. licheniformis* RG1 strains did not result in complete feather degradation^12^. Taken together, this evidence suggests that the adhesion of bacterial cells to the substrate surface plays an important role in the efficient and rapid hydrolysis of keratinous raw materials.

The mass spectrometric analysis of the secretory proteome of *B. paralicheniformis* T7 identified seven proteases, four of which belong to serine peptidase families S8 and S41, and three belong to metallopeptidase families M14, M42, and M55. Previously, 11 serine proteases of the S8 clan were established in the genome of *B. paralicheniformis* MKU3^35^. By the LC-MS/MS method, 2 extracellular serine proteases and 2 metalloproteases were established in *B. paralicheniformis* MKU3^36^, while 4 serine proteases and 3 metalloproteases were established in the secretory proteome of *B. paralicheniformis* T7. Notably, 2 proteases from *B. paralicheniformis* MKU3 with molecular masses of 87 kDa and 60 kDa correspond to the proteases S8 family serine peptidase and M14 family metallocarboxypeptidase detected in the secretory proteome of *B. paralicheniformis* T7. Further analysis showed that all identified serine peptidases and M14 carboxy metallopeptidase had signal peptides 28–36 amino acids in length. A specific contribution to the keratinolytic activity of this strain is likely attributable to an enzyme identified as an S8 serine peptidase 378 amino acids long with a molecular mass of 41.2 kDa. Sequence alignment revealed that this enzyme has high homology with the KerA keratinase from *B. licheniformis*^73^. A high score indicated that this peptidase was highly expressed in the secretory proteome of *B. paralicheniformis* T7.

Submerged *B. paralicheniformis* T7 fermentation on feather medium in a bioreactor revealed that under the specified conditions, the bacteria efficiently produced proteolytic enzymes after 7 h of fermentation. After 24 h of fermentation, protease and keratinase activities reached a maximum. Comparison with other bacillary strains (Table 7) showed that *B. paralicheniformis* T7 cultured on feather medium had production properties similar to those of *Bacillus* sp. CL33A^74^ but requires less time for fermentation. A short fermentation cycle was observed for *B. paralicheniformis* strain MKU3^25^, which also showed a maximum keratinase production at 24 h of fermentation.

**Table 7:** Comparison of the production properties of *Bacillus* strains during submerged fermentation.

Strain	Substrate	Incubation time (h)	Protease activity (U/mL)	Reference
*B. paralicheniformis* T7	Whole feathers and yeast extract	24	249.1	This work
*Bacillus* sp. CL33A	Whole feathers	216	248.0	^72^
*B. subtilis* KT004404	Malt extract and dextrose	48	55.1	^3^
*B. subtilis* PCSIR-5	Soybean meal	48	107.0	^73^

Feather medium is inexpensive and effective for protease production, and supplementation of the basic substrate with yeast extract or peptone positively affects the production properties of bacillary strains^74–76^.

Freeze-drying is a common but time-consuming method for obtaining concentrated enzyme preparations. However, the ability of *B. paralicheniformis* T7 proteases to withstand heating at 50–60 °C for up to an hour or more enables the preparation of concentrated proteolytic preparations by vacuum evaporation and spray-drying in heated air. Protease and keratinase activities in the concentrate, lyophilizate, and spray-dried powders were 4,940 U/mL, 148,670 U/g, and 193,670 U/g and 2,493 U/mL, 83,670 U/g, and 83,170 U/g, respectively. Overall, *B. paralicheniformis* T7 fermentation and the concentration and drying of proteolytic enzymes indicate the potential of *B. paralicheniformis* T7 as a producer of thermostable proteases and keratinases. Further studies on cloning the corresponding genes and producing recombinant proteases and keratinases for characterization and determining their individual and synergistic involvement in protein substrate hydrolysis would be of interest.

## Conclusion

This study describes a soil strain, *B. paralicheniformis* T7, with high proteolytic and keratinolytic activities. The enzymatic extract of this strain contains thermostable alkaline proteolytic enzymes from the serine peptidase and metallopeptidase families with distinct keratinase activity. Culturing *B. paralicheniformis* T7 on keratinous raw materials indicated that the strain completely degraded chicken feathers in 4 days and cattle hide in 7 days. The degradation rates for horns, hooves, and wool for this period were 3.6, 29.6, and 34.5%, respectively. SEM data showed that *B. paralicheniformis* T7 cells were highly and tightly adhered to the feather surface, accelerating the degradation process. Proteomic and genomic studies identified seven peptidases in the bacterial extract. Submerged fermentation of *B. paralicheniformis* T7 on feather raw material for 24 h yielded a protease activity of 249.1 U/mL. The milk-clotting activity of the strain was determined, in addition to its protease and keratinase activities. These results demonstrate that *B. paralicheniformis* T7 has the potential to produce thermostable proteolytic and keratinolytic enzymes. Future studies will aim at focusing on each identified protease and keratinase enzyme extract of the strain.

## Supplementary Information

Supplementary Material 1

## Data Availability

The complete *B. paralicheniformis* T7 genome has been deposited in NCBI GenBank under accession number CP124861 (https://www.ncbi.nlm.nih.gov/nuccore/CP124861). The authors declare that all data supporting the findings of this study are available within the article.
