# Supplementary material for: New Bacillus paralicheniformis strain with high proteolytic and keratinolytic activity
Source: Sci Rep. 2024 Sep 30;14:22621. doi: 10.1038/s41598-024-73468-8 (PMC11444040; doi:10.1038/s41598-024-73468-8)
Supplement: Supplementary file 1 — Supplementary Material 1 [file 41598_2024_73468_MOESM1_ESM.docx]

| **a**  1 2 3 4 5  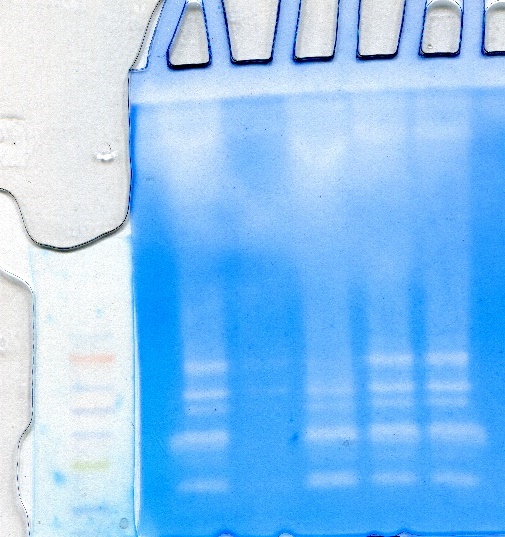 | **b** 1 2 3 4 5  **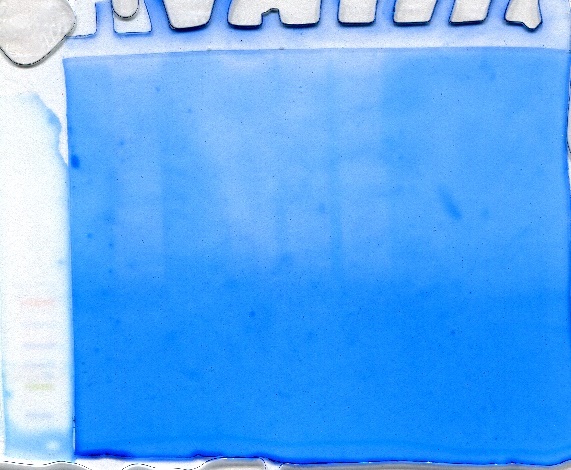** | **M**  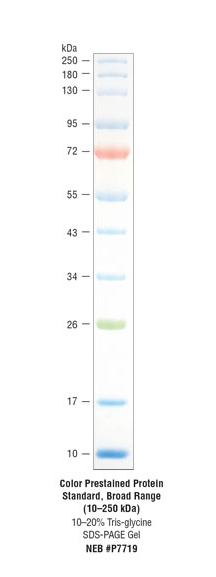 |
| --- | --- | --- |
| **c** 1 2 3 4 5  **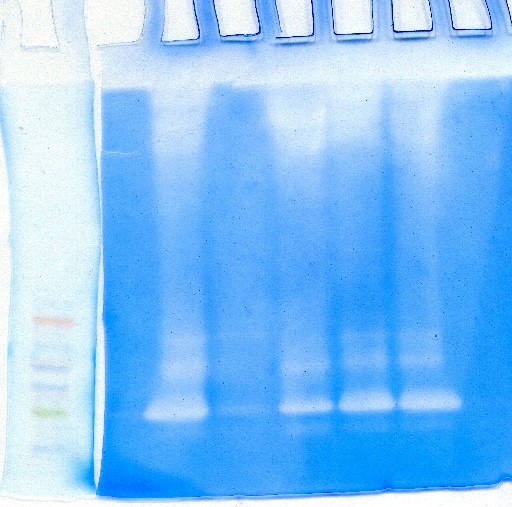** | **d** 1 2 3 4 5  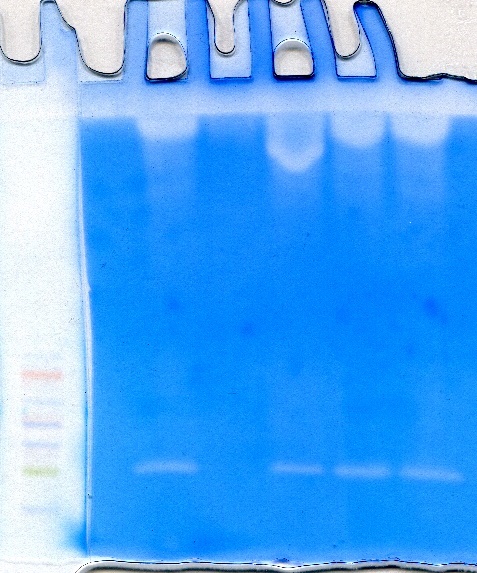 | 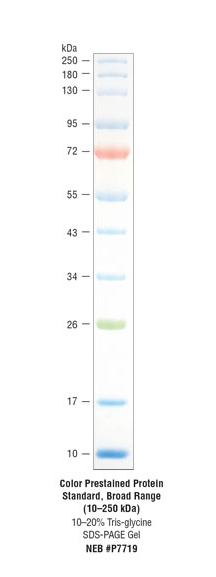 |

Supplementary figure S1. Zymogram with copolymerized casein (a), keratin (b), gelatin (c), and BSA (d) for the enzyme extract of B. paralicheniformis T7. Enzymatic extract (lane 1), enzymatic extract with PMSF (lane 2), enzymatic extract with EDTA (lane 3), enzymatic extract with Pepstatin A (lane 4), and enzymatic extract with E64 (lane 5). M – protein ladder.


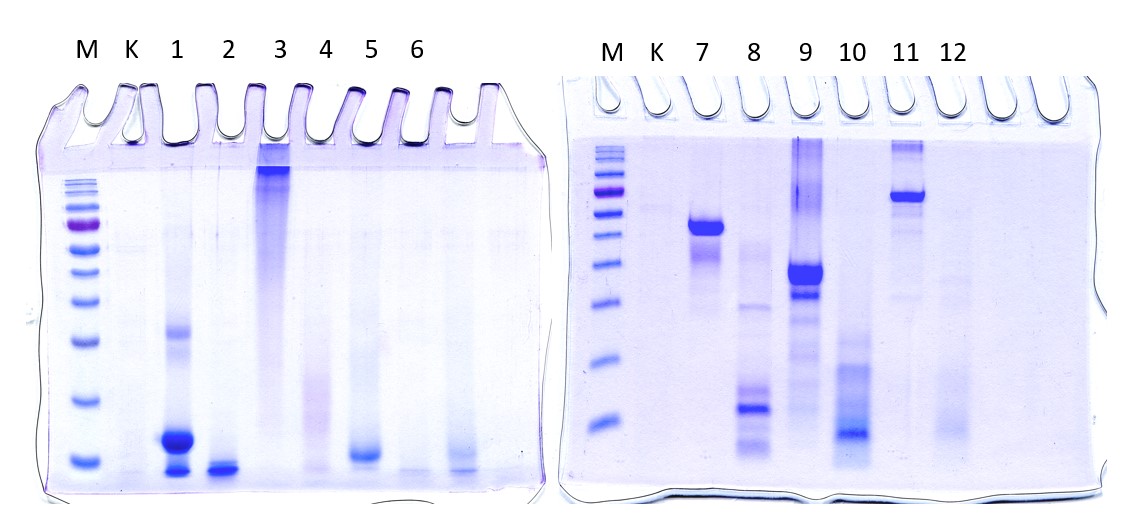


Supplementary figure S2. Hydrolysis of the proteins by enzyme extract of *Bacillus paralicheniformis* T7: М – protein marker NEB cat.#P7719S; K – enzymatic extract; 1 – hemoglobin, 2 – hydrolyzed hemoglobin in 5 min, 3 – gelatin, 4 – hydrolyzed gelatin in 5 min, 5 – keratin, 6 – hydrolyzed keratin in 30 min, 7 – ovalbumin, 8 – hydrolyzed ovalbumin in 1 min; 9 – casein; 10 – hydrolyzed casein in 15 sec; 11 – BSA; 12 – hydrolyzed BSA in 5 min.
